# An Unusual Case of a Double Tricuspid and Mitral Valves Infective Endocarditis Complicated by Multiple Septic Embolisms Secondary to an Atrial Septal Defect: A Case Report and Review of Literature

**DOI:** 10.3390/idr15050049

**Published:** 2023-09-04

**Authors:** Caterina Monari, Daniele Molinari, Alessandro Cornelli, Loredana Alessio, Francesco Coppolino, Consiglia Barbareschi, Stefania De Pascalis, Michele Torella, Giovanni Cimmino, Marisa De Feo, Nicola Coppola, Tiziana Formisano

**Affiliations:** 1 Infectious Diseases Unit, Department of Mental Health and Public Medicine, Section of Infectious Diseases, University of Campania Luigi Vanvitelli, Via L. Armanni 5, 80131 Naples, Italy; caterina.monari@gmail.com (C.M.); alessandro.cornelli@gmail.com (A.C.); loredana.alessio@gmail.com (L.A.); stefaniadepascalis77@tiscali.it (S.D.P.); 2 Cardiology Unit, Azienda Ospedaliera Universitaria Luigi Vanvitelli, Piazza Miraglia 2, 80138 Naples, Italy; daniele.molinari@gmail.com (D.M.); consiglia.barbareschi@unicampania.it (C.B.); giovanni.cimmino@unicampania.it (G.C.); tiziana.formisano@libero.it (T.F.); 3 Department of Women, Child and General and Specialized Surgery, Section of Anaesthesiology, University of Campania Luigi Vanvitelli, Piazza Miraglia 2, 80138 Naples, Italy; francesco.coppolino@unicampania.it; 4 Department of Translational Medical Sciences, Section of Cardiac Surgery and Heart Transplant, University of Campania Luigi Vanvitelli, via L. Bianchi c/o Ospedale Monaldi, 80131 Naples, Italy; michele.torella@unicampania.it (M.T.); marisa.defeo@unicampania.it (M.D.F.); 5 Department of Translational Medical Sciences, Section of Cardiology, University of Campania Luigi Vanvitelli, via L. Bianchi c/o Ospedale Monaldi, 80131 Naples, Italy

**Keywords:** multiple valve endocarditis, right- and left-sided endocarditis, endocarditis team, cardiac surgery

## Abstract

Multivalvular endocarditis (MVE) is an uncommon infection that mostly involves mitral and aortic valves, and it is related to a higher risk of congestive heart failure and a higher mortality. We described a case of a bilateral MVE and performed a review of the literature on similar clinical cases. We reported an unusual case of a 68-year-old male patient with a tricuspid and mitral infective endocarditis due to a methicillin-resistant *Staphylococcus aureus* complicated by multiple right- and left-sided septic embolization (lungs, brain, spleen, L2–L3 vertebral bones) due to an unknown atrial septal defect identified and repaired during cardiac surgery. Despite the severity of the clinical case, the patient experienced a good clinical outcome also thanks to a multidisciplinary approach. We identified 21 case reports describing bilateral MVE. A multidisciplinary approach is essential in the management of valve diseases to improve the prognosis of patients, especially in bilateral MVE.

## 1. Introduction

Infective endocarditis (IE) is a severe infection of the endocardium and heart valves. Despite remarkable improvements in diagnostics, therapeutic and microbiological tools, IE is still associated with a significant morbidity and mortality [1,2], with a 30-day mortality rate up to 30% [3]. In the last decades, the overall incidence has been rising worldwide, with substantial heterogeneity according to gender, age, and region [2]. The age-standardized incident rate has increased from 9.91 per 100,000 population in 1990 to 13.80 per 100,000 population in 2019 [2]. These findings may be due to several reasons, mainly the increase in life expectancy, a higher exposure to healthcare setting and higher frequencies of invasive procedures [3,4,5,6]. However, despite an increased complexity of IE clinical cases, a recent prospective cohort study by Ambrosioni et al. described an improvement in prognosis with a significant decrease in 6-month mortality [7].

The most common pathogen causing IE is *Staphylococcus aureus* (SA), responsible for 26–28% of cases, followed by oral *Streptococci* (12–18%) and non-oral *Streptococci* (5–17%) [3,4,5,6]. IE affects left-sided cardiac valves in 85–90% of cases and right-sided valves only in 5–10% of cases, mostly in people who injected drugs (PWID) or patients with dialysis or chemotherapy catheters [1,3,4]. Embolic events, i.e., the dissemination of endocarditic vegetations, are a frequent and life-threatening complication of IE, occurring in 20–50% of patients. Up to 25% of patients have embolic complications at the time of diagnosis [8]. The most frequent sites of embolic dissemination are the central nervous system and spleen in left-sided IE and lungs in right-sided and pacemaker-lead IE [8]. Multivalvular endocarditis (MVE) is uncommon; it mostly involves mitral and aortic valves, and it is related to a higher risk of congestive heart failure and a higher mortality [9,10].

A multidisciplinary approach, with the presence of an Endocarditis Team (ET), is essential in the management of valve diseases to improve the outcome of patients, especially in MVE [7,8,11,12].

We report an unusual case of a 68-year-old male patient with a tricuspid and mitral IE complicated by multiple right- and left-sided septic embolization due to an unknown atrial septal defect identified during cardiac surgery.

## 2. Case Report

We report the clinical case of a 68-year-old male who was admitted to a secondary-level hospital in October 2022 because of a 1-month history of low-grade fever (maximum temperature 37.8 °C), lumbar back pain, and a suspected intestinal occlusion, for which he underwent an exploratory laparotomy. In his past medical history, he reported a bladder cancer in 2016 but no cardiovascular risk factors. In the postoperative period, the patient developed high-grade fever (spikes above 38 °C), and three blood cultures (BCs) were performed and resulted positive for a methicillin-resistant *Staphylococcus aures* (MRSA) with the detection of *mecA* gene (minimum inhibitory concentration, MIC: oxacillin > 2 µg, vancomycin ≤ 0.5 ug, daptomycin ≤ 0.5 ug, ceftaroline 1 ug). The source was not detected. An antibiotic therapy with 1 g of vancomycin *bid (bis in die)* was started, and a transthoracic echocardiogram (TTE) was performed, revealing a 12 mm vegetation on the posterior leaflet of the tricuspid valve, determining a moderate tricuspid regurgitation. The left ventricle had normal dimension and preserved ejection fraction (EF, 55%); the right atrium and ventricle were mildly enlarged with preserved contractile function. A thorax CT scan described multiple pulmonary lesions compatible with septic emboli at the superior lobes. Therefore, the patient was transferred to our Infectious Disease Unit in November 2022.

On the day of admission in our unit, the patient was afebrile, confused, and reported low back pain without neurological signs or sensory deficits. No peripheral septic embolisms were detected during clinical examination. Vital parameters were stable, heart rate (HR) 84/min, blood pressure (BP) 120/67 mmHg, and a mitral murmur was heard. Blood exams revealed an increase in white blood cells count (WBC) (15,480/mm^3^) and in inflammatory marker (C-reactive protein, CRP, 24 times the upper limit of normal, uln). We decided to optimize the antibiotic treatment starting a combination therapy with daptomycin 10 mg/kg daily and ceftaroline 600 mg *tid (ter in die)*. The BCs collected at the admission (5 days after the start of vancomycin) confirmed the positivity for a MRSA strain as well blood cultures taken 72 h after the escalation of therapy. All the following blood cultures, performed at a 48 h interval, resulted negative. The presence of two major Duke’s criteria made the diagnosis of definite native tricuspid IE [8].

**Figure 1:**
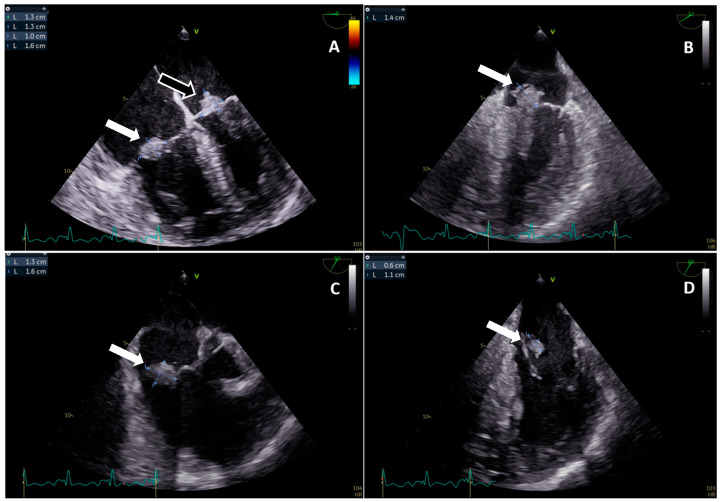
(**A**–**D**). Transoesophageal echocardiography performed in our hospital. (**A**): Mid-esophageal four-chamber view at 0° in TOE showing a 16 × 10 mm isoechoic vegetation on tricuspid posterior leaflet (white arrow) and another 13 × 13 mm isoechoic vegetation on mitral posterior leaflet (black arrow). (**B**): Mid-esophageal off axis view at 30° in TOE showing an isoechoic 14 mm vegetation on posterior mitral leaflet. (**C**,**D**): Mid-esophageal mitral commissural view at 60° in TOE showing an isoechoic vegetation on atrial side of mitral P3 scallop in different cardiac cycles (systole (**C**) and diastole (**D**)).

For a more accurate evaluation, a transoesophageal echocardiography (TOE) was performed and showed, beside a large (18 × 10 mm) and mobile vegetation adherent to the posterior leaflet of tricuspid valve determining a moderate-to-severe regurgitation, another mobile and isoechoic vegetation adherent to the atrial side of the posterior leaflet of mitral valve (12 × 16 mm) with a moderate regurgitation (Figure 1). No further peri-valvular complications were described. In the diagnostic work-up, we performed a cerebral magnetic resonance imaging (MRI), which revealed numerous cardio-embolic ischemic *foci* in the acute/subacute phase without signs of hemorrhagic infarction, a lumbar spine MRI with contrast, which showed an inflammatory process affecting the L2–L5 tract, and an abdomen computer tomography (CT) scan with contrast, which described multiple hypodense areas in the spleen compatible with splenic infarcts. A total body 18-F FDG PET-CT scan was also performed and showed a high uptake of the tracer at the tricuspid (SUV max 7) and mitral (SUV 5.4) valves as well as at L2–L3 vertebral bones (SUV 7.2).

The case was discussed by the local “endocarditis team” (ET), a multidisciplinary team composed by infectious diseases (ID) specialists, cardiologists, and cardiac surgeons. Considering the persistent vegetation despite the targeted antibiotic therapy, the multiple embolic events, and the acceptable surgical risk of the patient (EUROSCORE II 4.93%), the ET gave indication for surgery.

On 11 January 2023, the patient was then transferred to the cardiac surgery department, and a few days later, he underwent a double mitral and tricuspid valve replacement with bio-prosthesis (St Jude Medical, SJM (SJM, Inc, St. Paul, MN, USA), Epic Supra Valve 29 for the mitral, and an SJM Epic Supra Valve 33 for the tricuspid valve). During surgery, cardiac surgeons detected and closed a previously unknown atrial septal defect, which probably caused the tricuspid and mitral endocarditis and, thus, the multiple septic right- and left-sided embolisms. Post-surgery follow-up echocardiograms showed a normal functioning of both bio-prostheses with no regurgitation nor interatrial shunts. A new thorax CT scan showed the resolution of the previously described pulmonary septic *emboli*. We continued IV antibiotic therapy for 8 weeks until we received the negative result of the cultural exam of the explanted valves, and then we switched to an oral combination with trimethoprim/sulfamethoxazole 160/800 mg *tid* and doxycycline 100 mg *bid* for the treatment of lumbar spondylodiscitis. The patient was then transferred in a rehabilitation center in good clinical condition with normal vitals and blood exams (WBC 7420/mm^3^, CRP 1.7 uln).

The patient’s informed consent was obtained for the publication of the clinical case, and the study was approved by the Ethics Committee of the University of Campania L. Vanvitelli, Naples (n°17122/2023).

## 3. Review of the Literature

We performed a systematic review of the literature applying the search strategy in the Medline electronic database up to May 2023. The search strategy included the combination of two main domains (Text or Medical Subject Headings, MeSH): “bilateral infective endocarditis”, OR “right and left infective endocarditis”.

We included all the papers that met the following criteria: (1) Clinical cases on bilateral MVE; (2) Papers published as full text in English. We excluded studies with the following characteristics: (1) Cases of patients < 18 years old; (2) Mural EI or inter-atrial or -ventricular septum EI, or bilateral EI as complication of a monovalvular EI; (3) Reviews, meta-analysis, study protocols, retrospective or prospective studies, interim reports on prospective cohort, unpublished literature, and poster abstracts; (4) Papers reporting duplicated data.

**Figure 2:**
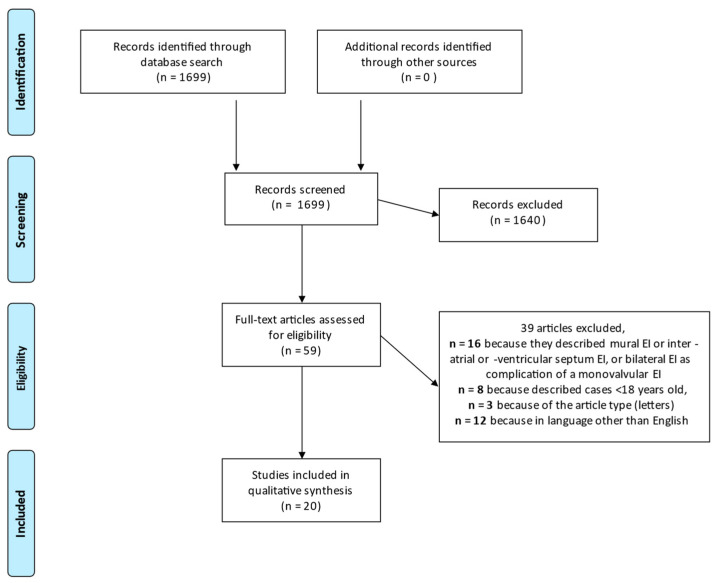
Flow-diagram of studies included in the review.

We identified 1699 studies reporting data on MVE (Figure 2). Among them, 20 papers reported 21 clinical cases on bilateral MVE [13,14,15,16,17,18,19,20,21,22,23,24,25,26,27,28,29,30,31,32].

Table 1 describes demographic, clinical, and microbiological characteristics as well as the prognosis of the 21 clinical cases identified and of our case. Different predisposing factors were described, such as IVDU, valve replacement, and hemodialysis, but six subjects had no known risk factors (Table 1). As in our clinical case, the predisposing factor for bilateral MVE was represented by atrial or ventricular septum defects (ASD or VSD, respectively) in five patients, but in three of them, it was identified and corrected during cardiac surgery [23,24,28]. Only four patients were managed by a multidisciplinary team (ET), and 15 underwent cardiac surgery. Five patients did not survive, and one experienced a long-term *sequela* due to dilatative cardiomyopathy and brain septic embolization (Table 1).

## 4. Discussion

We report an unusual clinical case of a 68-year-old man with a double native mitral and tricuspid IE complicated by multiple right- and left-sided septic dissemination *foci* due to MRSA. The cause of this unusual double right and left endocarditis was a previously unknown atrial septal defect, which was detected and fixed only during cardiac surgery.

Multivalvular endocarditis is a rare and severe condition with a poor prognosis [15,16,22,33,34,35,36]. A retrospective analysis of the Spanish Registry, with data from 2008 to 2020, reported a prevalence of MVE of 14.2% (577/4064 cases of IE) [37] with a mitral–aortic involvement in 87.9% of patients (507/577). Compared with patients with single-valve IE, patients with MVE experienced a higher rate of heart failure (42.7% vs. 52.9%, *p* < 0.001), in-hospital mortality (26.9% vs. 34.3%, *p* < 0.001) and need for surgery (67.7% vs. 85.1%, *p* < 0.001). Moreover, MVE was an independent factor of in-hospital mortality (odds ratio, OR, 1.3; 95% confidence interval, CI, 1.1–1.7; *p =* 0.004), even if it was not associated with a higher 1-year mortality (OR 1.1; 95% CI 0.9–1.4; *p* = 0.43) [37]. Similar results were described in another retrospective study performed in France in a cohort of 1304 patients with left-sided native valve IE [38]. The authors reported an MVE in 19% of cases and in these patients described a higher rate of embolic events (*p* = 0.044), congestive heart failure (*p =* 0.016), perivalvular complications (*p* < 0.001), early surgery (*p* < 0.001), and of 30-day mortality (24.5% vs. 17.6%, *p* = 0.008). Moreover, in MVE, an early surgery was associated with a higher 10-year survival rate (79 ± 4% vs. 35 ± 6%, *p* < 0.001) compared to medical management only. At the multivariate analysis, MVE remained an independent predictor of 30-day mortality (adjusted OR 1.86; 95% CI 1.26–2.73; *p* < 0.001) and of long-term mortality (hazard ratio, HR 1.7; 95% CI 1.31–2.11; *p* < 0.001) [38].

From a pathogenetic point of view, MVE may be caused by a simultaneous infection of two valves or a sequential seeding of previously damaged valves during a persistent bacteriemia [37,39]. In left-sided endocarditis, aortic IE may be considered a predisposing factor for secondary mitral-valve infection, whereas mitral IE does not specifically predispose to aortic involvement [33,38]. In most cases, the mitral valve may be damaged by a jet of aortic regurgitation, resulting in the appearance of vegetations, perforation, or pseudoaneurysm of the anterior leaflet of the mitral valve [33,34,35,39,40,41]. Other proposed pathogenetic mechanisms are the formation of abscesses, that destroy the mitral anulus, with or without mitral regurgitation, or the so-called “mitral kissing vegetation” phenomenon, where large aortic vegetations prolapse and involve the anterior mitral leaflet during the diastolic phase, spreading the infection to the mitral valve [33,36,37,38].

However, bilateral (right and left) IE, such the clinical case we have reported, is a rarer condition. In the above-mentioned Spanish registry, a bilateral involvement was identified in only seven patients (0.17%) of the 4064 enrolled [37]. Bilateral IE may be related to congenital heart diseases or defects, intra-cardiac devices, or repeated injections by drug users [22,37,41]. There are few cases of right- and left-sided MVE in the literature [13,14,15,16,17,18,19,20,21,22,23,24,25,26,27,28,29,30,31,32]. In addition to our patient, we have found in the literature another 21 clinical cases, which are outlined in Table 1. Patients were mainly males, with different predisposing factors, but six of them had no known risk factors. As described above, bilateral MVE is characterized by a higher need for surgery and a higher in-hospital mortality.

We assume that in our patient, the infection due to MRSA affected first the mitral valve and subsequently the tricuspid valve through the small interatrial defect detected during cardiac surgery. Surgery became necessary because of an uncontrolled infection despite the targeted antibiotic therapy and because of the isolation of MRSA, which is a very virulent and resistant bacteria with a low likelihood of being controlled by the sole antimicrobial therapy [8]. According to the European Society of Cardiology (ESC) guidelines, uncontrolled infection, defined as persisting infection and/or locally uncontrolled infection, i.e., increasing vegetation size, onset of abscess or pseudoaneurysms, is one of the most feared complications of IE and is the second most frequent cause for surgery [8].

Another topic suggested by the present clinical case is the importance of a multidisciplinary management of IE. In fact, because of its complexity and severity, IE is a disease that needs the collaboration of different professional figures, i.e., cardiologists, cardiac surgeons, infectious diseases specialists, microbiologists, neurologists, and neurosurgeons, in the so-called “endocarditis team”. In our center, all IE cases are discussed by the local endocarditis team. This approach is strongly recommended by the current guidelines [8,42], and it has been proven to be useful in optimizing the treatment strategy and in improving the prognosis of patients [7,9,10,43]. A before–after study performed in France in 2009 on 333 patients with IE described a significant decrease in 1-year mortality from 18.5% to 8.2% (hazard ratio, HR, 0.41; 95% CI, 0.21–0.79; *p =* 0.008) after the implementation of a multidisciplinary task force, which included recommendations on specimens sampling, medical therapy, duration of treatment, surgical indications, and a 1-year follow-up [10]. Another before–after study performed in 2013 reported similar results on patients affected by native IE after the introduction of a multidisciplinary strategy with a significant reduction in the overall in-hospital mortality (28% vs. 13%, *p* = 0.02) and 3-year mortality (34% vs. 16%, *p* = 0.0007) [9]. A more recent prospective study performed by Anguita Sánchez et al. on patients with IE over a period of 15 years described an increase in the elective surgery rates and a significant reduction in the overall in-hospital mortality (*p* < 0.01) after the implementation of a multidisciplinary strategy [43].

Thus, the multidisciplinary management thanks to the presence of the endocarditis team helped us obtain a favorable outcome in this severe case of unusual bilateral IE complicated by multiple right- and left-sided septic embolisms.

## 5. Conclusions

Bilateral EI is a severe infection characterized by a poor prognosis, a higher mortality rate and a higher need for surgical treatment. As in our clinical case, diagnosis may be challenging, and a multidisciplinary approach is of paramount importance. Moreover, beside the added value of an endocarditis team in terms of outcome, this peculiar clinical case underlines the additional importance of the combination of different aspects, such as clinical history, clinical examination, and diagnostic procedures in the management of IE.

## Figures and Tables

**Table 1:** Demographic, clinical, and microbiological characteristic of our clinical case and of MVE case reports in literature.

Author, Year [Reference]	Age, Years	Gender	Predisposing Factor	Etiology	Valve	ET	Right-Sided Embolism	Left-Sided Embolism	Surgery	Prognosis
Our clinical case	68	Male	Interatrial defect *	*Staphylococcus aureus MR*	Native TV and MV	Yes	Lungs	Brain, spleen, vertebral bones	Yes	Alive
Koshal 1981 [13]	55	Male	Dental extraction	*Streptococcus bovis*	Native AV and PV	No	NA	NA	Yes	Alive
Jeppson 2008 [14]	24	Female	None; elective absorption 10 days before diagnosis	*Streptococcus viridans*	Native AV and TV	No	\	\	Yes (2 times)	Dead (post-operatory complications leading to brain death)
Mutlu 2009 [15]	69	Female	Bio-prosthetic AV replacement	*Salmonella enteriditis*	Prosthetic AV; native MV and TV, right VW	No	\	\	Yes	Alive
van der Zee 2012 [16]	80	Male	Bio-prosthetic AV replacement (2 months before)	CoNS	Prosthetic AV; native MV and TV	No	\	\	Yes	Dead the day after surgery
	54	Male	None	*Staphylococcus aureus MS*	Native TV and MV	No	\	Splinter hemorrhage, Janeway’s lesions, arthritis	No	Alive
Jorge 2013 [17]	27	Male	VSD	Unknown	Native AV and TV, VSD	No	Lungs	\	Yes	Alive
Oylumlu 2013 [18]	26	Male	IVDU	*Staphylococcus aureus*	Native TV and MV	No	Lungs	\	No	Alive
Frey 2014 [19]	45	Female	None	*Streptococcus viridans*	Native AV and TV + aorto-cavitary fistula	No	\	\	Yes (2 times)	Alive
Birkenkamp 2015 [20]	63	Male	VSD	*Streptococcus anginosus*	Native MV and PV	Yes	Lungs	\	No	Alive
Khan 2015 [21]	36	Male	Bicuspid AV, previous IVDU, peripherally inserted CVC for 3 months	*Staphylococcus aureus (MS)*	Native AV, MV and TV	No	\	Heart block, fingers vasculitic lesions	Yes (3 times)	Alive
Sundaragiri 2015 [22]	31	Male	IVDU, previous MSSA IE (3 months before)	*Staphylococcus aureus MR*	Native TV and MV	No	Lungs	\	Yes	Dead after surgery
Daruwalla 2016 [23]	56	Female	HD via fistula, ASD *	*Staphylococcus aureus*	Native MV and TV	No	Lungs	\	Yes	Dead after surgery (sepsis)
Ishiekwene 2016 [24]	53	Male	VSD *	*Staphylococcus lugdunensis*	Native AV and MV	No	\	\	Yes	Alive
Fernando 2018 [25]	22	Female	VSD, bicuspid AV	*Streptococcus mitis*	Native MV and TV, right ventricular side of VSD	Yes	Lungs	Heart coronary	Yes	alive
Pan 2019 [26]	66	Male	None	*Streptococcus anginosus*	Native MV and TV	No	Lungs	\	Yes	Alive
Boyer 2020 [27]	57	Male	None	*Streptococcus mutans*	Native AV, PV, TV	No	Lungs	Brain	No	Alive with residual left-sided hemiparesis and dilated cardiomyopathy
Nemati 2020 [28]	54	Male	IVDU, VSD *	Cultures negative	Native TV and AV	No	\	Brain	Yes	Alive
Bolat 2021 [29]	62	Male	None	CoNS	Native MV and TV	No	\	\	Refused	Dead
Perez-Viloria 2022 [30]	66	Male	Congenital pulmonary stenosis	*Streptococcus mitis*	Native AO and PV	Yes	\	\	Yes	Alive
Tomoaia 2022 [31]	58	Male	ESRD on HD, previous *Ps. aeruginosa* TV EI (1 month before)	*Pseudomonas aeruginosa*	Native TV and AV	Yes	\	Brain, spleen	Yes	Alive
Haliga 2023 [32]	73	Female	Mechanical prosthetic MV (10 years before)	*Enterococcus faecalis*	Native AV, prosthetic MV	No	\	\	No	Alive

*: previously unknown. MS methicillin-susceptible, MR methicillin-resistant, CoNS coagulase-negative *Staphylococci*, AV aortic valve, MV mitral valve, TV tricuspid valve, PV pulmonary valve, VW ventricular wall, ET endocarditis team, IVDU intravenous drug user, ESRD end-stage renal disease, HD hemodialysis, ASD atrial septum defect, VSD ventricular septum defect.

## Data Availability

The data can be requested from the corresponding author, N.C. (nicola.coppola@unicampania.it).

## Abbreviations

MVE multivalvular endocarditis, IE infective endocarditis, MRSA methicillin-resistant *Staphylococcus aureus*, MSSA methicillin-susceptible *Staphylococcus aureus*, CoNS coagulase-negative *Staphylococci*, BC blood culture, ET endocarditis team, ESRD end-stage renal disease, HD hemodialysis, IVDU intravenous drug user, MIC minimum inhibitory concentration, TTE transthoracic echocardiogram, TOE transoesophageal echocardiogram, EF ejection fraction, MRI magnetic resonance imaging, PET-CT positron emission tomography-computed tomography, WBC white blood cells, CRP C-reactive protein, OR odds ratio, HR hazard ratio, AV aortic valve, MV mitral valve, TV tricuspid valve, PV pulmonary valve, VW ventricular wall.
